# Using Foreign Virtual Patients With Medical Students in Germany: Are Cultural Differences Evident and Do They Impede Learning?

**DOI:** 10.2196/jmir.6040

**Published:** 2016-09-27

**Authors:** Jens Walldorf, Tina Jähnert, Norman B Berman, Martin R Fischer

**Affiliations:** ^1^Universitätsklinik und Poliklinik für Innere Medizin IUniversitätsklinikum HalleMartin-Luther-Universität Halle-WittenbergHalleGermany; ^2^Department of PediatricsGeisel School of Medicine at DartmouthHanover, NHUnited States; ^3^Institut für Didaktik und Ausbildungsforschung in der Medizin am Klinikum der Universität MünchenLudwig-Maximilians-UniversitätMunichGermany

**Keywords:** virtual patients, medical education, cultural differences, competency-based education, e-learning

## Abstract

**Background:**

Learning with virtual patients (VPs) is considered useful in medical education for fostering clinical reasoning. As the authoring of VPs is highly demanding, an international exchange of cases might be desirable. However, cultural differences in foreign VPs might hamper learning success.

**Objective:**

We investigated the need for support for using VPs from the United States at a German university, with respect to language and cultural differences. Our goal was to better understand potential implementation barriers of a intercultural VP exchange.

**Methods:**

Two VPs were presented to 30 German medical students featuring a cultural background different from German standards with respect to diagnostic and therapeutic procedures, ethical aspects, role models, and language (as identified by a cultural adaptation framework). Participants were assigned to two groups: 14 students were advised to complete the cases without further instructions (basic group), and 16 students received written explanatory supplemental information specifically with regard to cultural differences (supplement group). Using a 6-point scale (6=strongly agree), we analyzed the results of an integrated assessment of learning success as well as an evaluation of cases by the students on usefulness for learning and potential issues regarding the language and cultural background.

**Results:**

The German students found it motivating to work with cases written in English (6-point scale, 4.5 points). The clinical relevance of the VPs was clearly recognized (6 points), and the foreign language was considered a minor problem in this context (3 points). The results of the integrated learning assessment were similar in both groups (basic 53% [SD 4] vs supplement 52% [SD 4] correct answers, *P*=.32). However, students using the supplemental material more readily realized culturally different diagnostic and therapeutic strategies (basic 4 vs supplement 5 points, *P*=.39) and were less affirmative when asked about the transferability of cases to a German context (basic 5 vs supplement 3 points, *P*=.048).

**Conclusions:**

German students found English VPs to be highly clinically relevant, and they rated language problems much lower than they rated motivation to work on cases in English. This should encourage the intercultural exchange of VPs. The provision of supplemental explanatory material facilitates the recognition of cultural differences and might help prevent unexpected learning effects.

## Introduction

Training with virtual patients (VPs) is a useful e-learning approach in medical education, complementing traditional curricular training strategies [1,2]. Using case-based e-learning software, practical knowledge and competence can be gained in a realistic setting [3]. Studies demonstrate that training with VPs improves aspects of knowledge and clinical reasoning in medical education [4-6]. Still, uncertainties exist regarding the optimal design and implementation of VPs [7,8], and the curricular implementation of this e-learning feature is rather limited [9].

A reason for the still limited use of VPs might be that creating high-quality medical e-learning material is a highly complex procedure and requires considerable time and knowledge resources [10,11]. Thus, it may be worthwhile to share existing high-quality VP cases among several medical institutions. In fact, an international exchange of VP cases might be attractive [12]. While English is widely accepted as the universal scientific language in medicine, the use of English e-learning material in general and VP cases in particular is not widespread in Germany. Therefore, the international exchange of VP cases might be quite challenging—obviously with regard to language barriers, but possibly also regarding cultural or national peculiarities in diagnostic or therapeutic procedures and guidelines as well as ethical aspects. Little is known about the effect of these specifics on the intercultural exchange of VPs. It is possible that cultural differences have an adverse effect on motivation or—even more problematic—on knowledge acquisition directly. German students using VPs from the United States for medical education might not be aware of cultural differences and thus acquire knowledge that might be, or seem, incorrect in their German medical context. On the other hand, it is possible that the recognition of these differences improves the motivation to deal with these cases and learn from contrasting effects.

We hypothesize that providing a supplemental description of the linguistic and cultural differences in VPs helps students identify and accept the differences and possibly use them as a stimulus for learning.

In this prospective study among German medical students, we investigated students’ perception of virtual cases written in English and featuring a different cultural background. We also examined the effect of offering supplementary explanatory material on learning success, motivation, and recognition of differences.

## Methods

To study the effect of cultural differences in working with VPs, we used the CASUS case-based learning environment [13]. The VPs were developed in a US medical context [10,14] and delivered in English. The CASUS platform uses a linear navigation concept, including varied task types, expert comments for feedback, and an integrated learning assessment of students’ performance. In these intermittent assessments, the students are asked several questions in different formats (eg, multiple choice and free text) regarding diagnostic or therapeutic procedures. The learning environment allows recording the results of the learning assessments as well as the time on task to complete each step of a case.

One case from family medicine (*fmCase*) and one from internal medicine (*imCase)* were selected because they contain substantial cultural differences to German standards or procedures and were appropriately challenging for fifth-year German medical students. *fmCase* consisted of a 39-year-old male with epigastric pain, Mr. Rodriguez, and *imCase* was Mr. Ramirez, a 78-year-old man with fever, lethargy, and anorexia (see Multimedia Appendices 1 and 2 for details on the learning objectives each case).

Specifically, the *fmCase* presents a 39-year-old Latino immigrant with epigastric pain. In this patient, a *Helicobacter pylori*‒associated gastric ulcer is diagnosed and repeatedly treated with antibiotics according to the results of serum and stool tests. The resistance pattern and prevalence of *H. pylori* infection vary from region to region, resulting in different (national) guidelines regarding diagnosis (ie, timing of endoscopy) and treatment (eg, choice of antibiotics) [15,16].

The *imCase* involves a 78-year-old male who suffers from urosepsis complicated by mesenteric ischemia and ultimately dies. Here, the clinical management of sepsis and gastrointestinal bleeding and especially aspects of palliative care are of interest, which are clearly based on different cultural backgrounds. National recommendations, legislation, and regulations show international differences [17,18].

To facilitate the recognition and classification of cultural differences, a simple categorization of distinct features of VPs (cultural adaptation framework) was outlined. Accordingly, both VPs were screened for cultural differences using the following categories: (1) diagnostic procedures, (2) therapeutic procedures, (3) the professional role of the medical student or the physician (eg, interaction with the patient), (4) ethical principles, and (5) language (highly specific medical terms and abbreviations, units).

Based on the differences, we developed an explanatory worksheet (the supplement) for each case. In addition to a glossary of specific medical English terms, this supplement included a critical discussion of cultural differences in the cases, as well as information on the corresponding procedures within the German medico-cultural background.

We aimed to keep these instructions as short as possible (resulting in 736 words and 1207 words per instruction, respectively; see Multimedia Appendices 3 and 4 for the full text of instructions). In an initial test, 5 voluntary students commented on the supplemental material and the evaluation forms, which were revised accordingly.

We then instructed 14 voluntary students to complete the cases including the integrated learning assessments, allowing the use of textbooks, Web-based dictionaries, and unit converters (the basic group). Subsequently, 16 students were instructed to complete the same cases under the same conditions plus the supplementary material in German (the supplement group).

An evaluation form (using the software EvaSys V6.1, Electric Paper Evaluationssysteme GmbH) was developed and revised after initial testing. In this evaluation, we specifically addressed the recognition of cultural differences, in particular considering language, medical and ethical aspects, and the interaction with patients. Also, all students were asked to assess their own motivation to work with VPs in general and specifically in English. They used a 6-point Likert-like scale (with 1=“strongly disagree” and 6=“strongly agree”). The students in the supplement group were also asked to evaluate the usefulness and efficacy of the supplementary material. The students were also asked to comment on the cases using free text.

We processed the results using Microsoft Excel (2003) for statistical analysis. To describe the results in the following section, the median is given together with the mean and standard deviation, where appropriate. To test for statistical differences between the groups, the Mann-Whitney U test (for comparing ordinal responses of two groups) or the chi-square test (for comparing frequencies) were applied. After Bonferroni correction for multiple testing, statistical significance was defined as *P*<.006.

The ethics committee of the Medical Faculty of the Martin-Luther-University Halle-Wittenberg, Germany approved the project.

## Results

Applying the suggested cultural adaptation framework on the two VPs, we identified 67 cultural differences. We identifed 58 differences (87%) as medical language issues, ten differences as diagnostic proceedings (eg, the relevance of endoscopy in diagnosing epigastric pain or lower gastrointestinal bleeding), five differences as therapeutic proceedings (eg, 81 mg vs 100 mg acetylsalicylic acid in coronary heart disease), three differences regarding the professional role of the physician or the medical student (eg, when obtaining informed consent), and one difference regarding decision making in a palliative situation (ethical/legal aspects).

In total, 33 students volunteered to participate in this study. Three students did not complete the cases and thus were not included in this analysis. The participants were fifth-year medical students (10 male and 20 female students, mean age 24 years [SD 2]; see Table 1), and all of them spoke German as their first language. The majority (29/33, 96%) of students reported learning English at school and many (22/33, 73%) of them had studied English for more than 7 years (Table 1). One student reported that he took English lessons at university. Six students (20%) spent more than 6 months in a country where English is the first language. During the last 6 months, the majority of participants (24/33, 83%) read at least one medical paper in English, and 5 students (16%) read at least one English medical textbook. There were no significant differences between the two groups regarding age, medical education (according to the year of medical education), or English language skills (as determined by classes at school or University).

When working with the cases, 23 students used Web-based English-German dictionaries (all students 77%, basic 73%, supplement 81%), 2 students used a paper-based dictionary, 13 students also used other online resources, and 7 students did not use any resources in addition to the material provided with the cases or supplements.

The results of the integrated learning assessments did not differ between the two groups of students: the basic and supplement groups answered 53% and 52% of the question items built into the VPs correctly (Table 1).

**Table 1:** Characteristics and performance of participants (learning assessment and required time).

	Basic	Supplement	All	*P* value
Students, n	14	16	30	
Age in years, mean (SD), median	24 (1), 24	25 (2), 24	24 (2), 24	.29^a^
Semester, mean (SD), median	8.9 (1.7), 9	9.9 (1.3), 10	9.4 (2.6), 10	.07^a^
Learned English >7 yrs in school, %	60%	81%	73%	.29^b^
Used e-dictionary, %	73%	81%	77%	.52^b^
Learning assessment, % correct answers, mean (SD), median	53 (4), 57	52 (4), 52	52 (4), 55	.32^a^
Time required (log files from both cases) in minutes, mean (SD), median	121 (52), 119	134 (38), 133.5	128 (44), 124	.45^a^

^a^Mann-Whitney U test

^b^Chi-square test.

The results of the evaluation are summarized in Table 2. Statements 1-4 refer to problems and motivation issues due to language problems. The students were clearly able to understand the instructions to the cases (Statement 3) and only reported minor language issues that potentially detracted from the actual cases (Statement 1). The students agreed very much that the clinical relevance of the cases was very understandable (Statement 2) and they felt motivated by working with English VPs (Statement 4). The provision of supplemental material did not influence language issues or motivation in this setting.

Statements 5 and 6 address differences in diagnostic and therapeutic procedures and the student-patient interaction *.* The transferability of the cases to a German cultural background was considered possible by the basic students (Table 2, Statements 5-7), while supplement students were clearly more skeptical (Table 2, Statement 7).

Statements 8 and 9 (Table 2) were exclusively addressed by the supplement group. These students agreed that the additional supplement was helpful to identify cultural differences between the proceedings in the patient cases and the knowledge, which is imparted within a German cultural background. To a lesser extent, the supplemental material was considered helpful in working with the VPs in general.

The free text comments of the students were reviewed with respect to the identification of cultural differences. Three students specifically noted differences in diagnostic procedures, and 4 students noted differences in therapeutic procedures. One student commented that the role of the medical student in the case was different to the students’ role in Germany. Three students pointed out differences in ethical aspects (insurance and accounting matters in medical care for immigrants, aspects of palliative care). Eight students specifically complained that they had to struggle with the English language or specific medical terms or abbreviations, and 2 students had problems with technical aspects of the Internet (eg, specific link not working, unpleasant layout).

**Table 2:** Results of the evaluation after completion of the two virtual patient cases. The students rated statements on a scale ranging from 6 (strongly agree) to 1 (strongly disagree).

	Basic	Supplement	All	*P* value^a^
1. I was busier with language problems than with the analysis of the case.	2.5	3	3	.29
2. The clinical relevance of the cases was understandable.	6	6	6	.42
3. For me it was difficult to understand the technical instructions in English.	1.5	2	2	.35
4. For me it was motivating to work with a case written in English.	4.5	4.5	4.5	.66
5. Diagnostic and therapeutic strategies are different to the strategies and procedures I am aware of.	4	5	4.5	.39
6. Medical students interacted with the patients in a way that was different from the interaction I am aware of.	2	3.5	2	.13
7. The described medical situation can be readily transferred to practices in Germany.	5	3	4	.048
8. I realized differences more readily using the additional information in the supplement.	n/a	5	n/a	
9. The supplements were helpful in working with the cases.	n/a	4	n/a	

^a^*P* values were calculated using the Mann-Whitney U Test.

## Discussion

### Principal Findings

Knowledge of cultural differences in medicine and medical education is very important, especially when interacting and communicating with patients or colleagues with a different cultural background. Typically, the term “cultural differences” is used to describe aspects of the doctor-patient or doctor-doctor relationship and refers to different explanatory models of health and illness, different cultural values, cultural differences in patient preferences for doctor-patient relationships, racism/perceptual biases, and linguistic barriers [19]. In this context, the development of “cultural competence” in medical education is considered to be highly important in medical education [20,21].

The cultural adaptation framework based on the experience with the 2 VPs in our study demonstrates that the term “cultural differences” should refer not only to the doctor-patient-relationship, but also to differences in diagnostic and therapeutic procedures, which may be caused by economic, epidemiologic, and historical differences. As no comparable framework for cultural adaptation exists so far, the proposed categorization of cultural differences in this context can be useful in future research as well as in adapting actual VPs for cross-cultural use.

One important result of this study is that VPs written in English are well accepted by German medical students: the students reported that working with the VPs was motivating, and despite minor language-related issues, the clinical relevance was very well understood (Table 2, Statements 1-4). Because intrinsic motivation is an important driver of learning activities [22], these results are clearly encouraging when discussing the interinstitutional and international exchange and availability of VPs in medical education [12].

Interestingly, although the supplement students received a concise list with translations of medical terms and abbreviations, they reported language-related difficulties similar to the students in the basic group (Table 2, Statement 1). Students more readily relied on their familiar (Web-based) translation resources than on looking up terms in the prepared list: 23 of the 30 students used online dictionaries, with no significant difference between the basic and supplement groups. Considering that the majority of cultural differences in our VPs consisted of linguistic peculiarities, this might indicate that the simultaneous use of supplements and Web-based dictionaries results in redundancy and possibly cognitive overload [23]. In order to reduce the cognitive load, it seems advisable to reduce the help translating English medical terms, especially since the students are well able to help themselves through language problems.

Our results underline the importance of providing additional information on cultural aspects of VPs. Similar observations have been made when using TV dramas in medical education [24] *.* Williams et al described “unexpected learning outcomes” in this context, which are clearly related to cultural differences, and strongly recommend a “reflection component” when using TV dramas for medical education. In our study, this reflection component consisted of written supplemental information. Discussions in small groups might be more effective, but they are more demanding as well.

### Limitations

The students’ evaluation has to be interpreted with some caution, as the recruitment of the students (self-selective, not randomized sample: voluntary participation) might have resulted in a substantial selection bias. It cannot be excluded that above-average motivated students took part in this study.

### Conclusion

The study results support our hypothesis that providing a supplemental description of the linguistic and cultural differences in VPs might help students identify and accept those differences, especially with regard to cultural differences in general. The supplemental material regarding linguistic differences was considered less valuable. If students are allowed to work in their normal context (ie, working online with access to Web-based dictionaries), linguistic information should be restricted to avoid redundant information and cognitive overload. However, it should not be left to the students themselves to identify important cultural differences in this context. The unsupervised use of such cases in extracurricular or private study generally might be beneficial but problematic if certain aspects of cultural differences are not noticed by students. When recommending the use of foreign VPs, it would be advisable to ensure that the medical procedures, ethical principles, or role models taught in the cases fit to the knowledge and competences that are intended. Specific cultural differences should be identified and discussed. This might result in an intensified work-up of the cases and might possibly lead to a better understanding of the learning objectives.

## Multimedia Appendix 1

Learning objectives fmCASE.

## Multimedia Appendix 2

Learning objectives imCase.

## Multimedia Appendix 3

Supplemental material fmCase.

## Multimedia Appendix 4

Supplemental material imCase.

## Abbreviations

VP: virtual patient
