# Supplementary material for: Using Foreign Virtual Patients With Medical Students in Germany: Are Cultural Differences Evident and Do They Impede Learning?
Source: J Med Internet Res. 2016 Sep 27;18(9):e260. doi: 10.2196/jmir.6040 (PMC5059482; doi:10.2196/jmir.6040)
Supplement: Supplementary file 1 [file jmir_v18i9e260_app1.pdf]

MedU

fmCASES

Case 19

39-year-old male with epigastric pain - Mr. Rodriguez

Author: Joel Heidelbaugh, M.D., University of Michigan

Learning Objectives:

1. Create a differential diagnosis for a male patient who presents with epigastric abdominal pain.
2. Highlight key features of the history and physical examination that support the diagnosis of peptic ulcer disease.
3. Describe the differences in diagnostic workup and treatment for peptic ulcer disease due to Helicobacter pylori (H. pylori) versus gastroesophageal reflux disease (GERD).
4. Highlight the differences between ulcer and non-ulcer (functional) dyspepsia.
5. Outline evidence-based treatment strategies for H. pylori gastritis.
6. Discuss appropriateness of follow-up and testing for eradication in patients with H. pylori gastritis.
7. Provide an overview of potential risks and adverse events associated with non-judicious use of proton pump inhibitors (PPIs).
8. Learn basics about cultural competency and respect for patients who will require interpreter services.

Summary of Clinical Scenario: Mr. Rodriguez is a 39-year-old uninsured Latino immigrant with no significant past medical history who presents with a worsening pain in his upper abdomen over the last year, now occurring daily. His symptoms sometimes improve with meals and other times worsen with meals or with spicy foods. He denies vomiting, hematemesis, hematochezia, or melena as well as any general, cardiovascular, respiratory, and genitourinary symptoms. He occasionally takes ibuprofen when sore or tired after work. He recently quit smoking and consumes alcohol occasionally. His father has hypertension, and his mother has diabetes. His physical exam is unremarkable.

A differential diagnosis of abdominal pain is generated and Mr. Rodriguez is given a trial of omeprazole to test and treat his dyspepsia. Four weeks later, he returns with his symptoms unchanged. Fecal immunochemical testing (FIT) is performed

to check for occult gastrointestinal bleeding and is negative. Blood serum testing for H. pylori IgG is positive. He is given standard proton pump inhibitor (PPI) triple therapy to eradicate suspected H. pylori infection.Nine weeks later, Mr. Rodriguez returns to the clinic with no change in his symptoms. An H. pylori fecal antigen test is positive, and he is given salvage therapy. Two weeks later he reports he is symptom-free.

Key Findings from History	• 39-year-old Latino male<br>• Epigastric abdominal pain<br>• Symptoms improve and/or worsen with meals<br>• Lack of hematemesis<br>• Lack of hematochezia or melena<br>• Alcohol use
Key Findings from Physical Exam	• None
**Differential Diagnosis**	• Gastroesophageal reflux disease (GERD)<br>• Dyspepsia due to peptic ulcer disease (PUD)<br>• Anxiety<br>• Abdominal wall muscle strain<br>• Gastritis
Key Findings from Testing	• Fecal immunochemical testing (FIT): Negative<br>• *H. pylori* IgG assay: Positive<br>• *H. pylori* fecal antigen test (at 9-week follow-up visit): Positive
**Final Diagnosis**	• *H. pylori* ulcer

Case Highlights: This case highlights the use of interpreters and barriers to health care faced by such patient populations as the uninsured and minorities.

Key Teaching Points

Knowledge

Differential diagnosis of abdominal pain:

**Gastrointestinal**	• Appendicitis<br>• Cholecystitis<br>• Constipation and fecal impaction<br>• Diarrhea<br>• Diverticulitis<br>• Dyspepsia (e.g., peptic ulcer disease, non-ulcer dyspepsia, functional dyspepsia)<br>• Gastroesophageal reflux disease<br>• Acute or chronic hepatic failure with resultant complications (e.g., ascites)<br>• Hepatitis (e.g., viral, autoimmune)<br>• Inflammatory bowel disease<br>• Intestinal ischemia<br>• Intestinal obstruction<br>• Intractable nausea and vomiting<br>• Irritable bowel syndrome<br>• Pancreatitis<br>• Perforation (e.g., gastric, colonic, intestinal)<br>• Pyloric stenosis<br>• Tumor (e.g., gastric, hepatic, pancreatic, intestinal, colonic)
**Cardiac**	• Myocardial infarction<br>• Angina pectoris<br>• Abdominal aortic aneurysm dissection or rupture
**Psychogenic**	• Anxiety<br>• Panic disorder<br>• Post-traumatic stress disorder
**Pulmonary**	• Pleurisy<br>• Pneumonia<br>• Pulmonary infarction<br>• Tumor
**Renal**	• Nephrolithiasis<br>• Pyelonephritis<br>• Tumor<br>• Urinary tract infection

**Other**	• Abdominal wall muscle strain<br>• Hernia (e.g., inguinal, incarcerated)<br>• Metabolic (e.g., drug overdose, ketoacidosis, iron or lead poisoning, uremia)<br>• Psoas abscess<br>• Subphrenic abscess<br>• Trauma<br>• Dietary intolerance (lactose, fructose, artificial sweeteners)

Potential barriers to medical care for Latinos:

- Many Latinos, especially farm workers, are undocumented immigrants. They may fear that if they seek medical attention, the healthcare system may report them to the government, placing them at risk of deportation.
- Traditional Latinos view health from a holistic standpoint, where physical problems cannot be separated from nonphysical problems.
- Latinos are less likely to visit a physician's office or access preventive services, including vaccinations.
- Many Latinos, especially recent immigrants, may view the U.S. healthcare system as confusing, intimidating, and unfriendly. They may also believe that they receive lower quality of a medical care and lesser treatment standards because of discrimination and racism.
- The Latino culture places great emphasis on masculinity, and survey-based data has found that many Latino men avoid routine healthcare because they view themselves as "strong."

Dyspepsia:

Definition

- Literally, "bad digestion"
- Upper abdominal pain or discomfort that is episodic or persistent
- Often associated with belching, bloating, heartburn, nausea, or vomiting

Prevalence

- About 25% adults are affected by it; many self-diagnose and self-treat it
- Accounts for approximately 5% of all visits to family practitioners
- The most common reason for a referral to a gastroenterologist in the U.S.

Etiology

- Functional (non-ulcer) dyspepsia (NUD): No identifiable etiology (~50%)
- Peptic ulcer disease (PUD) (15–25%)
- GERD (5–15%)

- Gastric or esophageal cancer (<2%)
- Pancreatitis (rare)

Peptic ulcer disease (PUD):

Risk factors: No evidence to support a cause-and-effect association between acetaminophen, psychosocial stress, caffeine intake, or cigarette smoking and PUD.

Pharmacological agents:

- Aspirin
- Non-steroidal anti-inflammatory drugs (NSAIDs)
  - Chronic NSAID use is a leading cause of morbidity in the elderly
- Chronic anticoagulation with warfarin
- Chronic corticosteroid therapy

Cigarette smoking

- Decreases vascularity to gastric mucosal cells, resulting in decreased rates of mucosal healing after insult.

Moderate to severe physiologic stress

- Especially intensive care unit (ICU) patients

H. pylori infection (see below)

Gastroesophageal reflux disease (GERD):

Definition

- Reflux through the lower esophageal sphincter (LES) into the esophagus or oropharynx

Etiology

- Abnormal LES pressure and increased reflux during transient LES relaxations are believed to be key etiologic factors.

Precipitants

- Spicy and fatty foods and chocolate
- Smoking
- Drinking alcohol and caffeinated beverages
- Eating large portions
- Lying flat in close temporal proximity to a meal
- Wearing tight clothing around the waist
- Medications (calcium channel blockers, beta-agonists, alpha-adrenergic agonists, theophylline, nitrates, and some sedatives)

Atypical signs and symptoms

- When severe reflux reaches the pharynx and mouth or is aspirated, it can cause:
  - Asthma
  - Chronic cough
  - Dental enamel loss
  - Globus sensation
  - Hoarseness
  - Non-cardiac chest pain
  - Recurrent laryngitis
  - Recurrent sore throat
  - Subglottic stenosis

These symptoms may point to (but do not sufficiently support by themselves) a diagnosis of GERD.

Complications

- While GERD generally does not progress (up to 90% of cases of GERD are non-erosive reflux disease [NERD]), some cases are associated with development of complications.
- Symptom frequency, duration, and severity do not help to differentiate the grade of esophagitis and cannot be used to reliably diagnose complications of GERD.

Reported health-related quality of life is lower than age-matched patients who have untreated angina pectoris, diabetes mellitus, or chronic heart failure.

<u>Distinguishing dyspeptic symptoms of GERD and PUD:</u>

**Dyspepsia due to GERD**	**Dyspepsia due to PUD**
Classic symptoms of heartburn and regurgitation (burning in the chest with sour or bitter taste) clearly dominating patient's history indicates diagnosis of GERD with high specificity	Difficult to separate PUD from other etiologies of dyspepsia based on symptoms alone.
• Epigastric burning that sometimes radiates to the throat<br>• Esophageal spasm: Sharp, stabbing, substernal pain	• Episodic or recurrent epigastric "aching," "gnawing," or "hunger-like" pain or discomfort arising from the proximal gastrointestinal tract

Most likely to occur:<br>• When gastric volume is increased (after meals)<br>• When gastric contents are located near the gastroesophageal junction (due to recumbency or bending)<br>• When gastric pressure is increased (with obesity, pregnancy, binding clothes, or girdles)	There may be differences in timing of symptoms based on ulcer location:<br>• Gastric ulcer pain often occurs 5–15 minutes after eating and remains until the stomach empties, which may be up to several hours in duration; pain may be absent during times of fasting.<br>• Pain from duodenal ulcers is often relieved by eating, drinking milk, or taking antacids, and may return anywhere from 90 minutes to 4 hours after eating a meal.<br>• Both gastric and duodenal ulcers may be associated with nausea and vomiting occurring any time from shortly after eating to several hours later.

Complications of GERD and PUD:

Complications of GERD	Complications of PUD
• Esophagitis (develops when the mucosal defenses are overwhelmed by refluxed acid, pepsin, or bile)<br>• Peptic strictures from fibrosis and constriction (10%)<br>• Barrett's esophagitis (replacement of the squamous epithelium of the esophagus by columnar epithelium)<br>• 2–5% of Barrett's esophagitis may be further complicated by	• Hemorrhage<br>• Perforation into the peritoneal cavity or adjacent organs (causes severe, persistent abdominal pain)<br>• Ulcer scar healing or inflammation with impaired gastric emptying can lead to gastric outlet obstruction syndrome.

adenocarcinoma	

Alarm symptoms related to GERD or PUD: These symptoms require immediate referral of patient to a gastroenterologist.

**Symptom**	**Concern**
**Dysphagia (difficulty swallowing)**	Suggests development of peptic stricture; rapidly progressive dysphagia potentially indicates adenocarcinoma.
**Onset of heartburn and regurgitation in patient > 55 years old**	Increased chance of cancer
**Early satiety**	May be associated with gastroparesis or gastric outlet obstruction due to stricture or cancer
**Hematemesis (vomiting blood)**	Bleeding ulcer, mucosal erosions, esophageal tear (Mallory-Weiss tear), esophageal varices
**Hematochezia (passage of blood with stool)**	Rapidly bleeding ulcer or mucosal erosions
**Iron deficiency anemia**	May indicate possible bleeding from a peptic ulcer, GERD-related mucosal erosions, or Barrett's ulcer
**Odynophagia (painful swallowing)**	Associated with infections, erosions, or cancer

**Recurrent vomiting**	Suggestive of gastric outlet obstruction
**Weight loss**	Associated with malignancy

Helicobacter pylori (H. pylori) infection:

Prevalence

- Varies across geographic regions, ethnic groups, and household conditions
- Rare in developed countries, and worldwide prevalence is decreasing
    - § ri.
    - 80–90% of all adults, regardless of age, in developing nations (including Latin and Central America), are infected with H. pylori.
- Results of study of Latinos in San Francisco Bay Area:
    - Immigrants (31% infected with H. pylori)
    - First-generation U.S.-born Latinos (9%)
    - Second-generation U.S.-born Latinos (3%)
    - Both household and birth-country environment have probably contributed to declining H. pylori prevalence among successive generations of Latinos.

Transmission

- In underdeveloped nations, thought to occur fromfecal-oral transmission during childhood

Mechanism of action

- H. pylori is uniquely adapted to life in the stomach. Its location in the gastric mucosa, where it does not invade the gastric epithelium, provides the organism with protection from the host immune mechanisms, which creates challenges in the delivery of antimicrobial agents to eradicate infection.
- Colonization renders underlying gastric mucosa more vulnerable to peptic acid damage by disrupting the mucous layer, liberating enzymes and toxins, and adhering to the gastric epithelium.
- Host immune response to H. pylori incites an inflammatory reaction, which further perpetuates tissue injury.
- Chronic inflammation leads to chronic gastritis (in most cases, asymptomatic and nonprogressive).

Complications

- In some cases, altered gastric secretion coupled with tissue injury leads to PUD, while in other cases, gastritis progresses to atrophy, intestinal metaplasia, and eventually gastric carcinoma.
- Rarely, persistent immune stimulation of gastric lymphoid tissue leads to

gastric lymphoma.
- 90% of patients worldwide with duodenal ulcers are infected withH. pylori.
- The strongest evidence to support the role of H. pylori as an etiology of PUD is the elimination of ulcer recurrence after eradication.

Skills

History:
Conduct the practice of "medication reconciliation" at each encounter to determine the medications and doses patients are currently taking.

Use of an interpreter:

- Have interpreter sit unobtrusively to one side
- Speak directly to patient, not to interpreter
- Interpreter should be simply a conduit for the conversation

Physical exam:

In most cases with symptoms related to GERD and PUD, the physical examination will be normal. Look for signs of complications and other diseases that could be associated with dyspepsia:

- Hemodynamic status:Hypotension or tachycardia may indicate significant blood loss from a gastrointestinal bleed.
- Signs of anemia: Brittle nails and cheilosis (cracks and sores on the lips) are signs of anemia, and pallor of palpebral mucosa or nail beds may be present.
- Signs of malignancy: Weight loss, palpable mass, signal nodes (Virchow's nodes) and acanthosis nigricans (velvety, light-brown-to-black markings usually found on the neck, under the arms, or in the groin) are signs of possible malignancy.
- Signs of gallbladder disease: Jaundice or positive Murphy's sign (performed by applying pressure under the right costal margin and asking patient to take a deep breath. If patient stops inhaling [as tender gallbladder comes in contact with examiner's fingers], the test is considered positive.)
- Signs of hypo or hyperthyroidism: Cool, pale skin, coarse hair, or non-pitting edema (myxedema), or delayed relaxation phase of deep tendon reflexes (DTRs) may be present with hypothyroidism; diarrhea, warm skin, thinning hair, eyelid lag, brisk DTRs, and tachycardia may be present with hyperthyroidism. Both diagnoses should be considered in the evaluation of dyspepsia.

Differential diagnosis

1. **Anxiety**: May be associated with different types of body pain. Patients who

have anxiety disorders may self-medicate with alcohol, which may contribute to gastrointestinal disorders.

2. **Gastroesophageal reflux disease (GERD)**: May present with mild epigastric abdominal pain. Symptoms commonly worsen with meals.
   - Hematemesis in the setting of GERD represents an alarm symptom indicative of an upper GI bleed or tumor and warrants prompt GI referral for evaluation and upper endoscopy.
   - Hematochezia and melena are not typically associated with GERD.
3. **Abdominal wall muscle strain**: Commonly associated with upper abdominal and epigastric pain.
4. **Dyspepsia due to peptic ulcer disease (PUD)**: Epigastric abdominal pain that improves with meals is the hallmark of dyspepsia due to PUD. However, in some cases, symptoms may worsen with meals.
   - Hematemesis suggests more complicated disease, including bleeding ulcer, and warrants prompt GI referral and endoscopy.
   - Hematochezia and melena commonly occur in the setting of an upper GI bleed secondary to PUD.

Less likely diagnoses:

Acute pancreatitis: More likely associated with abrupt, stabbing abdominal pain (often radiating to the back), nausea, vomiting, appearing ill on exam, and clinical signs of dehydration, such as tachycardia. Epigastric pain associated with pancreatitis usually worsens with eating. Acute and chronic pancreatitis may be caused by alcohol use. Hematemesis in the setting of pancreatitis-like symptoms suggests an upper GI disorder (e.g., ulcer) that warrants hemodynamic stabilization and prompt GI referral. Hematochezia and melena are not typically associated with pancreatitis.

Pneumonia: Unlikely in the absence of pulmonary symptoms

Diverticulitis: Commonly presents with left lower quadrant abdominal pain, hematochezia, and fever. It is the most common cause of lower GI bleeding in patients > 50 years of age. More quiescent cases can be managed in the outpatient setting with trimethoprim/sulfamethoxazole and metronidazole or levofloxacin and metronidazole. More complicated cases are often admitted to the hospital for intravenous antibiotics and fluid resuscitation and may require surgical evaluation.

Angina pectoris: Classically presents with substernal chest pain, but may present with epigastric abdominal pain and nausea or vomiting. Interestingly, GERD is the most common cause of non-cardiac chest pain.

Studies

Evaluation of dyspepsia:

Upper GI series

- Useful in diagnosing complications of GERD (e.g., esophageal stricture)
- Poor utility in diagnosing GERD
- May reveal gastric or duodenal ulcer, but not considered the diagnostic gold standard

24-hour pH probe

- Most appropriately utilized when the diagnosis of GERD cannot easily be determined
- When patients desire referral for surgical treatment of their GERD/hiatal hernia (Nissen fundoplication)
- When patients with classic symptoms of GERD (heartburn, regurgitation) do not improve after appropriate trials of PPIs

Fecal immunochemical testing (FIT) and fecal occult blood testing (FOBT):

- Consider one of these tests if no improvement with PPI test-and-treat strategy
- FIT more sensitive and specific than FOBT for detecting occult lower GI bleeding
- FIT is not suitable for detecting gastric bleeding and should not be used if the suspected source of bleeding is proximal to the ligament of Treitz
- Guaiac-based FOBT (including Hemoccult II SENSA) best used to check for occult upper GI bleeding
- Multiple negative FOBTs or FITs do not exclude the presence of either upper or lower GI blood loss
- Diets high in red meat, iron, and vitamin C may cause false positive results with guaiac-based tests.
- Multiple negative FOBTs or FITs do not exclude presence of either upper or lower GI blood loss
- Diets high in red meat, iron, and vitamin C may cause false positive results in guaiac-based tests

Complete blood count: Useful to evaluate for anemia, but neither sensitive nor specific for GI bleeding

H. pylori testing: Should be performed only if clinician plans to offer treatment for positive results.

H. pylori IgG serologic test:

- A useful first-time test in population with high prevalence of active infection
- Confirms evidence of past infection and an immunologic response
- Cannot be used to confirm eradication of H. pylori after treatment

Urease breath test:

- Accurately detects active infection
- More expensive than serologic testing
- Less accurate during PPI therapy (stop PPI for at least 2 weeks and bismuth for at least 4 weeks prior to breath test)
- May be used as confirmatory test after positive serologic test

Fecal antigen testing:

- Accurate, but more expensive and less convenient than serologic testing and may not be available in all settings
- May be used to evaluate eradication after pharmacotherapy for H. pylori gastritis, which may be a more cost-effective strategy than performing a urease breath test

Management

GERD or PUD:

- A **test-and-treat strategy** for both GERD and PUD is the most widely accepted initial therapeutic intervention.
    - Often, patients begin a self-directed trial of over-the-counter anti-secretory therapy (AST): either a histamine-2 receptor antagonist (H2RA) or a PPI. Patients consult their primary care physicians because their symptoms have persisted or because they would like a prescription (to reduce out-of-pocket cost).
    - “PPI test” (short-term trial of a PPI)
        - Sensitive and specific for diagnosing GERD and can significantly reduce the need for upper endoscopy/EGD and 24-hour pH monitoring
        - Saves over $350 per patient evaluated, reduces upper endoscopies by 64%, and reduces the number of esophageal monitoring tests by 53%
    - Anti-secretory therapy should be stopped after a successful 4 to 8-week course, or used on demand (only when symptoms recur).
- Refer for **upper endoscopy/EGD** to rule out significant disease if:
    - There are alarm or extra-esophageal symptoms, or
    - In cases that do not respond to the test-and-treat strategy after 8 weeks
    - Endoscopy should include biopsies of gastric body and antrum to test for H. pylori
- **Lifestyle modifications** for patients with GERD (although there is little evidence to support improvement in symptomatic outcomes in the absence of pharmacotherapy):
    - Avoid large meals
    - Avoid acidic foods (citrus- and tomato-based products), alcohol, caffeinated beverages, chocolate, onions, garlic, and peppermint
    - Decrease dietary fat intake
    - Avoid lying down within 3–4 hours after a meal

- Avoid medications that may potentiate GERD symptoms, including calcium channel blockers, beta agonists, alpha-adrenergic agonists, theophylline, nitrates, and some sedatives
- Elevate head of bed 10 to 20 cm (4–8 inches)
- Avoid wearing clothing that is tight around the waist
- Lose weight
- Stop smoking

Functional dyspepsia: **Therapies** are **similar to those for PUD**.

- PPI therapy superior to placebo treatment with regard to relative risk reduction of functional dyspepsia (number needed to treat [NNT] = 9).
- Empiric trial of histamine-2 receptor antagonists may or may not be beneficial.
- A subset of patients with functional dyspepsia will not respond favorably to any form of anti-secretory therapy and will continue to experience symptoms.
- 10% reduction in dyspepsia symptoms 12 months after H. pylori eradication therapy in patients with functional dyspepsia (NNT = 17).

Therapies reserved for use after H. pylori infection is ruled out:

- Tricyclic antidepressants (a systematic review determined that there is insufficient evidence to support the efficacy of psychological therapies for the treatment of functional dyspepsia; however, individual trials have reported some modest clinical benefits in symptomatic improvement.)
- Alternative therapies: Capsaicin, peppermint oil, caraway oil, and artichoke leaf may improve symptoms. (Peppermint oil, however, decreases lower esophageal sphincter pressure and may worsen GERD symptoms in some patients.)
- Patients should be cautioned that herbal remedies are not regulated by the U.S. Food and Drug Administration (FDA), may not have been studied for safety, and can have adverse side effects.

Current recommended first-line regimens forH. pylori eradication:

- PPI triple therapy for 10–14 days (70–85% eradication rate):
  - PPI standard dose twice daily (esomeprazole is dosed once daily), plus
  - Clarithromycin 500 mg twice daily, plus
  - Amoxicillin 1 gram twice daily
- Alternative PPI triple therapy for 14 days (70–85% eradication rate): (Consider in patients with penicillin allergy.)
  - PPI standard dose twice daily, plus
  - Clarithromycin 500 mg twice daily, plus
  - Metronidazole 500 mg twice daily
- Quadruple therapy for 10–14 days (75–90% eradication rate)
  - PPI standard dose once or twice daily OR ranitidine 150 mg twice daily, plus
  - Tetracycline 500 mg 3 times daily, plus

    - Metronidazole 250 mg 4 times daily, plus
    - Bismuth subsalicylate 525 mg 4 times daily

Accepted indications for testing to prove H. pylorieradication after antibiotic therapy:

- Patients with H. pylori-associated ulcers
- Individuals with persistent dyspeptic symptoms despite the test-and-treat strategy
- Individuals with H. pylori-associated MALT (mucoid-associated lymphoid tissue) lymphoma
- Post-resection of early gastric cancer
- If needed to document clearance in patients planning to resume chronic NSAID therapy

To evaluate eradication of H. pylori:

- Perform fecal antigen testing
- If fecal antigen test positive:
    - Patient requires re-treatment with salvage therapy for a resistant infection
    - If symptoms continue to persist:
        - Upper endoscopy/EGD to rule out ulcer disease and obtain mucosal biopsy for evaluation of persistent H. pylori infection
        - Prolonged PPI therapy for symptomatic control
- If fecal antigen testing negative (or unavailable) and the patient continues to have symptoms:
    - Perform urease breath testing
    - If urease breath testing negative, patient should be referred to a gastroenterologist for upper endoscopy/EGD and mucosal biopsy for evaluation of persistent H. pylori infection

Back to top

- Copyright © 2011 iInTIME. All Rights Reserved.
-
