# Supplementary material for: Using Foreign Virtual Patients With Medical Students in Germany: Are Cultural Differences Evident and Do They Impede Learning?
Source: J Med Internet Res. 2016 Sep 27;18(9):e260. doi: 10.2196/jmir.6040 (PMC5059482; doi:10.2196/jmir.6040)
Supplement: Supplementary file 2 [file jmir_v18i9e260_app2.pdf]

MedU

simple

Case 21

78-year-old man with fever, lethargy, and anorexia – Mr. Ramirez

Author: Joseph T. Wayne, M.D. Albany Medical College

Learning Objectives

1. Distinguish among the types of shock and their presentations.
2. Discuss the common causes for and symptoms of lower gastrointestinal (GI) blood loss.
3. Recommend appropriate empiric therapy for urosepsis based on an understanding of urinary tract infection pathogenesis and resistance patterns.
4. List elements of physical exam in patient with suspected GI bleed.
5. Take an accurate blood pressure.
6. Interpret a urinalysis.
7. Recommend laboratory and diagnostic tests to evaluate GI bleeding.
8. Understand the physician's role when a patient is no longer capable of making medical decisions.

Summary of clinical scenario: A 78-year-old man is admitted with three days of fever, lethargy, and anorexia. He initially rallies, but later suffers a lower gastrointestinal bleed and declines.

Key Findings from History	• Fever<br>• Anorexia and weight loss<br>• Progressive weakness<br>• Mental status changes<br>• History of abdominal aortic aneurysm repair
Key Findings from Physical	• Fever<br>• Hypotension<br>• Tachycardia

	• Tachypnea<br>• Mental status changes (otherwise negative neurological exam)<br>• No localizing signs of infection
**Differential Diagnosis**	• Metabolic disorder<br>• Meningitis<br>• Sepsis
Key Findings from Testing	• Leukocytosis with left shift<br>• Elevated BUN:creatinine ratio<br>• U/A: positive for nitrite, leukocyte esterase, WBC's<br>• PT/INR and PTT: elevated<br>• Hgb: decreasing<br>• Lactic acidosis<br>• CT of the abdomen: segmental bowel-wall thickening and pneumatosis; cecal mass; liver metastases
**Final Diagnosis**	• Urosepsis, mesenteric ischemia, metastatic colon cancer

Case highlights: Follows a complex patient with multiple medical issues through his hospital course, including transitioning to a palliative care approach. Students learn the treatment for sepsis and acute GI bleeding and how to counsel patients regarding advance directives.

Key Teaching Points

Knowledge

Systemic inflammatory response syndrome (SIRS):
Definition: An inflammatory state without a proven source of infection (vs. **sepsis**: systemic inflammation with a suspected or proven infection). Two of the following four cardinal signs are required:

- Temperature > 38.3 C (101.0 F) or < 36 C (96.8 F)
- Pulse > 90 beats/min or < 45 beats/min
- Respiratory rate > 20 breaths/min or PaCO2 < 32 mm Hg
- WBC > 12,000 cells/mm3, < 4000 cells/mm3, or with 10% immature forms

Etiology: Believed to be caused by cytokine dysregulation. Can occur in following

settings:

- Severe trauma
- Burns
- Acute pancreatitis
- Surgery

Sepsis: Systemic inflammation with a suspected or proven infection

Severe sepsis: Sepsis with acute end-organ dysfunction as evidenced by any of the following:

- Ileus (absent bowel sounds)
- Acute oliguria (urine output < 0.5 mL/kg/hr for at least two hours)
- Hypoxemia
- Thrombocytopenia
- Rise in serum creatinine > 0.5mg/dL
- Coagulation abnormalities (INR > 1.5 or PTT > 60 seconds)
- Bilirubin > 4mg/dL (68 μmol/L)
- Unexplained metabolic acidosis

Additional signs/symptoms may include:

- Altered mental status
- Hyperglycemia
- Elevated plasma C-reactive protein (> 2 standard deviations above normal)
- Elevated plasma procalcitonin (> 2 standard deviations above normal)

Septic shock: Acute circulatory failure with persistent arterial hypotension as defined below and unexplained by other causes:

- Systolic blood pressure of < 90 mm Hg
- Mean arterial pressure (MAP) < 60 mm Hg, or
- Reduction in systolic blood pressure of > 40 mm Hg from baseline, despite adequate volume resuscitation

Urinary tract infection (UTI): While gram-positive organisms account for most cases of sepsis, gram-negatives account for most UTI-associated sepsis:

- 70 to 80%: community-acquired gram-negative pathogens (e.g., E. coli, Klebsiella, and Proteus)
- Other possible organisms:
    - Citrobacter and Pseudomonas
        - Gram-negative pathogens associated with hospitalization or instrumentation
    - Enterococci
        - Appear as gram-positive cocci in chains
        - Would raise concern about the integrity of gastrointestinal mucosa

  - Staph saprophyticus
    - Found almost exclusively in females; much less common in men due to length of the urethra)
    - Staph species appear as gram-positive cocci in clusters
  - Group B Streptococcus
    - Sometimes seen in patients with diabetes

Causes of gastrointestinal (GI) bleeds:
Upper GI bleeds (i.e., above ligament of Treitz)

- Erosive esophagitis
- Varices
- Gastritis
- Ulcers (gastric and duodenal)

Lower GI bleeds (i.e., below ligament of Treitz and almost always from the colon; jejunal or ileal sources of bleeding are very rare)

- Ulcerative colitis
- Diverticulosis
  - Presence of multiple diverticuli (small pockets in the colon due to defects in the wall)
  - Distinct from diverticulitis, when diverticuli fill with stagnant fecal material setting up the inflammatory cascade and possibly causing obstruction or perforation
  - Bleeding usually occurs in a non-inflamed diverticulum (i.e., in the setting of diverticulosis)
- Infectious colitis
- Colon cancer

In the elderly also consider:

- Angiodysplasia (degenerative or congenital structural abnormality of the vasculature)
- Mesenteric ischemia (ischemia or infarction of the small bowel and left colon)
  - Usually underlying atherosclerotic cardiovascular disease
  - History of prolonged (one to two hours), post-prandial abdominal pain (intestinal angina), weight loss due to poor absorption, and, frequently, fear of eating due to the expected pain
  - When acute, a catastrophic event
  - Pain often out of proportion to physical findings of distention, nausea, and vomiting
- Ischemic colitis
  - Prolonged hypotension and decreased perfusion affect blood supply to right colon via inferior mesenteric artery
  - Also usually underlying atherosclerotic cardiovascular disease
  - Little or no pain
  - Once circulation is restored, bleeding is self-limited

    - Recovery often complete

Skills

History:
Anorexia (loss of appetite); seen with:

- Depression
- Malaise
- Febrile illnesses
- Gastrointestinal disorders
- Dementia
- Alcohol and drug addiction
- Side effect of medication

Hematochezia: Passage of visible blood in the stool
Melena: Black, sticky, tarry stools

Physical exam:
GI exam
Document presence or absence of distension, tenderness to palpation, rebound tenderness, guarding, bowel sounds. Perform rectal exam, obtaining stool sample to check for evidence of bleeding in GI tract.
Altered mental status

- Delirium
    - Relatively acute decline in cognition that fluctuates over hours or days
    - Hallmark: deficit of attention
    - All cognitive domains are variably involved:
        - Memory
        - Executive function
        - Visuo-spatial tasks
        - Language
    - Associated symptoms
        - Altered sleep-wake cycles
        - Perceptual disturbances (e.g., hallucinations or delusions)
        - Affect changes
        - Autonomic findings (e.g., heart rate and blood pressure instability)
- Drowsiness
    - Defined as not perceiving the environment fully and responding to stimuli appropriately, but slowly or with delay
    - Patient may be roused by verbal stimuli but may ignore them
    - Patient is capable of verbal response unless aphasia, aphonia, or anarthria is present
    - Lethargy is a form of drowsiness

Blood pressure measurement

- Cuff should have a bladder length that is 80% and a width that is at least 40% of arm circumference (i.e., length-to-width ratio of 2:1)
- Error is minimized with a cuff width of 46% of the arm circumference
- Recommended cuff sizes:

Arm circumference	Cuff size
22 to 26 cm	"small adult" (12 X 22 cm)
27 to 34 cm	"adult" (16 X 30 cm)
35 to 44 cm	"large adult" (16 X 36 cm)*
45 to 52 cm	"adult thigh" (16 X 42 cm)*

*In these sizes the ideal width ratio of 46% of arm circumference is not practical, because it would result in a width of 20 cm and 24 cm, respectively—not clinically usable for most patients.

- Because the bladder is enclosed in the cuff, it is difficult for the clinician to know the length.
- No manufacturers' standards for the different sizes
- Recommended to label individual cuffs with the ranges of arm circumferences to which they can be correctly applied (preferably by having lines that show whether the cuff size is appropriate when it is wrapped around the arm)
- Morbidly obese patients may have very large arm circumferences with short upper arm length (difficult to correctly cuff, even using thigh cuff). Alternative techniques:
  - Place cuff around forearm and listen for sounds over radial artery (may overestimate systolic blood pressure)
  - Use validated wrist blood pressure monitor held at heart level

Differential diagnosis

1. **Cardiogenic shock**: Associated with acute coronary syndromes and characterized by acute pulmonary edema, elevated jugular venous pressure (JVP).
2. **Hypovolemic shock**: Typically a history of hemorrhage or volume loss (diarrhea or polyuria).
3. **Neurogenic shock**: Associated with spinal cord or severe central nervous system injury and findings of bradycardia and hypotension.
4. **Anaphylactic shock**: Associated with antigenic exposure. Presents with urticaria, angioedema, and wheezing.

5. **Adrenal crisis**: Often presents with abdominal pain, nausea, vomiting, weakness, lethargy, hypotension, and skin pigmentation changes.
6. **Septic shock**: Initially with high cardiac output, fever or hypothermia (< 96.8 F), tachycardia (> 90 beat/min), tachypnea (RR > 20 breaths/min). Commonly will present with confusion or delirium and a suspected source of infection.

Studies

Complete blood count (CBC) with differential:

- Evaluates for leukocytosis (present in ischemia or infection)
    - Also may indicate a "left shift" (term remaining from the days when white blood cells were counted manually, under a microscope: the total number of bands—the less mature form of neutrophils—was written down first, on the left-hand side of the laboratory report; today, the term "left shift" means that the bands, or stabs, have increased, indicating an infection in progress)
- Leukopenia can also indicate a poor prognosis
- Low platelet count might indicate disseminated intravascular coagulation (DIC)
- Peripheral smear helpful if microangiopathic hemolytic anemia suspected (would see schistocytes and helmet cells)
- Also helpful in determining blood loss (acute blood loss may not be evident on CBC until after IV fluid is given)

Chemistry panel:

- Necessary to evaluate for metabolic derangement
- When physical exam indicates dehydration, need to know electrolytes and renal function to properly administer intravenous fluids:
    - Hypernatremia can be seen in dehydration (a possibility when the patient is confused and has inadequate free water intake)
    - Renal failure may be diagnosed by an elevated creatinine
    - Hyperkalemia often seen in renal failure
    - Elevated BUN:creatinine ratio (normal is between 6 and 20) suggests dehydration with pre-renal failure
- Elevation of hepatic transaminases (AST/ALT) may indicate liver involvement
- Calculation of the anion gap:
    - Na - (chloride + bicarbonate)
        - Normal ≤ 12
        - Elevated anion gap indicates metabolic acidosis caused by various ingestions, lactic acidosis from poor organ perfusion (e.g., bowel ischemia), diabetic ketoacidosis, or significant uremia (very elevated BUN)

PT/PTT:

- Elevation of these values indicates abnormalities in the coagulation cascade
- Seen in disseminated intravascular coagulation (DIC) and sepsis

Urinalysis:

- Can be done quickly
- Examination of sediment can determine presence of:
  - White blood cells (WBCs), indicating infection
  - WBC casts, indicating pyelonephritis
  - Red blood cells (RBCs) and RBC casts, indicating possible glomerulonephritis
- Gram stain of an unspun specimen can confirm presence of bacterial infection as well as morphology of the offending bacteria and help direct appropriate therapy
- Specific gravity aids in confirming dehydration
- Hyaline casts can also be seen in dehydration

Blood and urine cultures:

- Isolate causative organism
- Demonstrate appropriate sensitivities to direct antibiotic therapy

Erythrocyte sedimentation rate (ESR):

- Most likely elevated in setting of infection
- If cultures are negative, or if suspicion for vasculitis, an elevated ESR (> 50 mm/hr) may be helpful

Clostridium difficile toxin assay: Infection with Clostridium difficile should be considered in hospitalized patients exposed to antibiotics

- Typically does not develop for at least 48 hours after initiation of antibiotic therapy
- Produces marked thickening of the colon on CT scan as well as very high total white blood counts

Type and screen:

- Should be performed on any patient who may need to be transfused with blood products (such as packed RBCs)

Type and cross:

- Minimizes risk of transfusion reactions by matching patients' blood type and potential antibodies to donor blood
  - Some hospitals prefer to obtain type and screen first as, once type and cross is done, blood will be wasted if not administered to patient.

Plain abdominal x-ray:

- Good initial imaging choice for severe abdominal pain, but frequently

non-specific
- Helpful in ruling out perforation (seen as free air under the diaphragm) or obstruction (seen as air-fluid levels)
- Distention or pneumatosis (gas in the bowel wall) can be seen in advanced ischemic colitis

Abdominal CT scan:

- Not very useful for acute GI bleeding
- If ischemic colitis or obstruction is suspected, may confirm obstruction or show pneumatosis (bowel-wall thickening in a segmental pattern) and gas in the mesenteric veins (consistent with ischemic colitis or mesenteric ischemia)
- Perform without contrast if patient's renal function is impaired

Electrocardiogram (EKG): Although considered routine, an EKG is not absolutely necessary. Would be helpful if signs/symptoms of endocarditis, myocardial ischemia, or rhythm disturbance.

Chest x-ray (CXR): Not necessary if history is not concerning for a pulmonary process and respiratory rate and O2 saturations are normal

Colonoscopy:

- Consider if diagnosis remains unclear after CT scan (only if no clinical or radiologic evidence of peritonitis or perforation)
- Pseudomembranous colitis is seen as yellowish round plaques and membranes
- Can biopsy colon to determine underlying pathophysiology (e.g., inflammatory bowel disease)
- With suspected or known lower GI bleed, perform colonoscopy within 48 hours of onset (therapeutic procedures such as electrocautery or injection with epinephrine can be performed at time of procedure)
- Ischemic colitis may show pale mucosa with petechial bleeding; cyanotic mucosa in more severe disease
    - For bleeding refractory to colonoscopic intervention, surgical consult should be obtained for possible resection of the ischemic segment

Management

Restoring volume to prevent organ damage

- Give fluid bolus of 10 to 20 cc/kg of normal saline (NS) or lactated Ringer's (LR) over thirty minutes (depending on patient status and associated conditions.
    - Can give smaller bolus when associated conditions such as congestive heart failure or chronic kidney disease are present.
- NS and LR are isosmotic and will provide immediate restoration of

intravascular volume while also providing tissue rehydration.

Antibiotic treatment:

- Administer antibiotics within one hour of determining the potential source of infection and after all appropriate cultures are obtained.
- Antibiotic choice should be based on local sensitivities reported through the infectious disease tracking system at each hospital (antibiogram).
- Ampicillin: Effective treatment; however, high resistance rates preclude its use as empiric first-line therapy
- Trimethoprim/sulfamethoxazole (TMP/SFX): Resistance rates against this agent vary and make it, too, less desirable as first-line treatment
- Possible empiric agents:
    - Third and fourth generation cephalosporins (have broad gram-negative and partial gram-positive coverage)
    - Piperacillin/tazobactam
    - Ticarcillin/clavulanate
    - Impenem
    - Meropenem
    - Aztreonam
- Aminoglycoside: Not a good choice of empiric drug (primarily due to its nephrotoxicity), but consider if Pseudomonas is a possibility or patient is immunocompromised

Once organism is identified and sensitivities are known, antibiotic therapy can be more specifically directed toward offending pathogen.

Monitor urine output:

- Insertion of Foley catheter
- Can adjust fluids according to output and vital signs

RBC transfusion:

- Common to reserve transfusions for patients with hemoglobin < 8 g/dL (100 g/L) with exception of patients with cardiovascular disease (transfuse if hemoglobin < 10 g/dL)
- Can monitor low hemoglobin with serial CBCs every 6 hours

Medical decision-making:

- Physicians assess decision-making capacity as a part of routine clinical care.
- Any physician (not only a psychiatrist) has authority to determine if patient has decision-making capacity for a specific medical decision.
    - However, only a court can determine if a patient is "competent" (a legal term) to make a decision
    - When courts determine competency, they rely on state laws that define incapacity (different according to state)
    - Physicians do not determine if patients are competent but often testify

at competency hearings

- A surrogate decision-maker can be identified to make medical decisions on behalf of a patient who is determined to be incapable of making a medical decision.
- Thirty-one states have laws listing a legal hierarchy of surrogate decision-makers. In Washington state, for example, the order is as follows:
  1. Court-appointed guardian
  2. Durable power of attorney (DPOA) for health care
  3. Spouse (if patient is married, spouse becomes the legal surrogate, unless s/he has completed a DPOA for health care or the court has appointed a legal guardian)
  4. Adult children
  5. Parents
  6. Adult siblings

Back to Top

- Copyright © 2012 iInTIME. All Rights Reserved.
-
