# Supplementary material for: Using Foreign Virtual Patients With Medical Students in Germany: Are Cultural Differences Evident and Do They Impede Learning?
Source: J Med Internet Res. 2016 Sep 27;18(9):e260. doi: 10.2196/jmir.6040 (PMC5059482; doi:10.2196/jmir.6040)
Supplement: Supplementary file 3 [file jmir_v18i9e260_app3.pdf]

Hilfestellung zur Bearbeitung des fmCASES 19
“39-year-old male with epigastric pain - Mr. Rodriguez”

Card 1

preceptor	Lehrer

Card 2

ER	emergency room
GI	gastrointestinal
GU	genitourinary
CVS	cardiovascular system

Card 3

Yerba Buena	allgemeine Bezeichnung für Heilkräuter

Card 8

GI	gastrointestinal, hier: Gastroenterologe
Acetaminophen	Paracetamol

Card 11

98.5 Fahrenheit = 36.9° Celsius

pallor	Blässe
rales or rhonchi	Rasselgeräusche

Card 12

In den USA kostet eine Ösophagogastroduodenoskopie im Mittel 1600 €, in Deutschland etwa 50 €. Das beeinflusst natürlich auch die Entscheidungsfindung zur „rationellen“ Diagnostik.
In Deutschland wäre es durchaus denkbar, den 39-jährigen Patienten zur endoskopischen Diagnostik beim Gastroenterologen vorzustellen – wenngleich in der Regel ein Behandlungsversuch wie im Fall vorgeschlagen erfolgen wird. Ältere Patienten (über 45 Jahre) oder Patienten mit höherem Risiko (Alkohol-, Nikotin- und Familienanamnese) sollten in jedem Fall endoskopisch untersucht werden, da die Wahrscheinlichkeit einer bösartigen, zugrunde liegenden Erkrankung (Ösohaguskarzinom) mit dem Alter steigt.

Card 14

Vgl. Kommentar zu Card 12. Dementsprechend wäre in der im Fallbeispiel beschriebenen Situation eine Überweisung zum Gastroenterologen durchaus angemessen.

Card 15

complete blood count with platelets: Blutbild (im deutschsprachigen Bereich schliesst das sog. „kleine Blutbild“ die Plättchenzahl (Thrombozytenzahl) stets ein. Das „große

Blutbild“ oder „Diff-BB“, Differentialblutbild, umfasst eine Differenzierung der Leukozyten (neutrophile, eosinophile, basophile Granulozyten, Lymphozyten und Monozyten).

FOBT Faecal occult blood test, fäkaler ookulter Bluttest (z.Bsp. Hämoccult) – Stuhlgujaktest, detektiert Häm im Stuhl

Modernere Test detektieren immunologisch Globin und weisen eine höhere Sensitivität und Spezifität auf (zum Beispiel Fecal Immunochemical Testing (FIT), and immunochemical fecal occult blood test (iFOBT)), sind allerdings kostenintensiver.

Card 16

Die Prävalenz der H.p.-Infektion variiert mit der geographischen Verteilung, der ethnischen Zugehörigkeit und dem sozioökonomischen Status. Sie zeigt eine altersabhängige Zunahme. Eine Verbesserung des Lebensstandards führt zu einem Rückgang der Prävalenz der H.p.-Infektionen. Die Infektion wird von Mensch zu Mensch übertragen, meist von Eltern auf ihre Kinder.

In Deutschland beträgt die Prävalenz bei Kindern 5%, bei Erwachsenen 24%, deutlich höher ist sie bei Immigranten (Kinder 36-44%, Erwachsene 52-86%).

Für eine zuverlässige Diagnostik sollten zwei Tests auf H.p. positiv sein (ausser bei einem Ulcus duodeni, das in >90% mit H.p. assoziiert ist - hier reicht aufgrund der hohen Vortestwahrscheinlichkeit für das Vorliegen einer H.p.-Infektion eine Testung aus). Die doppelte Testung ist aufgrund der niedrigen und fallenden Prävalenz von H.p.-Infektionen in Industrieländern notwendig (bei sinkender Prävalenz sinkt auch der positiv prädiktive Wert).

Bei Patienten mit nicht endoskopisch untersuchten, anhaltenden dyspeptischen Beschwerden wird gemäß deutschen Leitlinien die alleinige H.p.-Testung mit anschliessender Eradikationsbehandlung („test & treat“) nicht empfohlen. Diese Strategie ist nur sinnvoll in Ländern mit höherer H.p.-Prävalenz (wie im Fallbeipsiel), insbesondere, wenn die Kosten für eine Endoskopie hoch sind, und die endoskopische Diagnostik nicht breit verfügbar ist. In den USA kostet eine Ösophagogastroduodenoskopie im Mittel 1600 €, in Deutschland etwa 50 €.

Card 17

Problematisch bei der antibiotischen Therapie von H.p. ist insbesondere die Clarithromycin-Resistenz (die Resistenz gegen Amoxicillin ist vergleichsweise sehr gering und nicht entscheidend in der Therapieauswahl). Weltweit ist die Clarithromycin-Resistenz von 9% im Jahr 1998 auf über 17% im Jahr 2008 gestiegen, in Industrieländern kann die Häufigkeit dieser Resistenz > 20% betragen. Insofern hängt die Therapieauswahl von der lokalen Resistenzlage ab. In Deutschland liegt die Clarithromycin-Resistenz < 20%, so dass in erster Linie die erwähnte Therapie mit Protonenpumpenhemmern, Clarithromycin und Amoxicillin empfohlen werden kann. Die Vierfachtherapie ist unüblich.

Card 21

Da es sich um eine Famulatur im Bereich family medicine (hausärztliche Versorgung) handelt, kann auf eine „Anbindung“ des Patienten an Ihre Praxis eine sinnvolle Empfehlung sein. In einer gastroenterologischen Sprechstunde wäre eine Wiedervorstellung nur bei Beschwerden erforderlich, eine weitere ärztliche Betreuung durch einen Allgemeinmediziner (bzw. „Hausarzt“) sollte unter Versorgungsaspekten dennoch empfohlen werden.

Card 22

Nationale Leitlinien Ulcus ventriculi, Helicobacter pylori und gastroösophageale Refluxerkrankung (GERD)
