# Supplementary material for: Using Foreign Virtual Patients With Medical Students in Germany: Are Cultural Differences Evident and Do They Impede Learning?
Source: J Med Internet Res. 2016 Sep 27;18(9):e260. doi: 10.2196/jmir.6040 (PMC5059482; doi:10.2196/jmir.6040)
Supplement: Supplementary file 4 [file jmir_v18i9e260_app4.pdf]

**Hilfestellung zur Bearbeitung des imCase 21**
**“78-year-old man with fever, lethargy, and anorexia – Mr. Ramirez”**

Card 1

*Internal Medicine Rotation* entspricht etwa dem Tertial Innere Medizin im Praktischen Jahr.

anorexia = Inappetenz

Card 2

Der Kontakt mit Patienten ohne ausreichende Deutschkenntnisse ist auch bei uns nicht ungewöhnlich. Oft findet sich im Personal der eigenen Abteilung oder „nebenan“ jemand, der als Dolmetscher einspringen kann – wie auch hier.

ED = emergency department (Notaufnahme)

Card 3

| | |
|---|---|
| osteoarthritis | Arthrose |

81 mg Aspirin täglich sind in Deutschland eine zur Thrombozytenaggregationshemmung (Erhaltungsdosis) ungebräuchliche Dosierung, typischerweise werden in Deutschland 100 mg ASS tgl. verordnet. Allgemein wird zur Sekundärprävention kardiovaskulärer Ereignisse die Einnahme von 75 – 100 mg ASS tgl. empfohlen, bei Vorhofflimmern bis 325 mg tgl.

| | |
|---|---|
| Acetaminophen | Paracetamol |
| prn. | pro re nata (bei Bedarf) |

Card 4

| | |
|---|---|
| VS | Vital Signs |
| HR | Heart Rate, d.h. die eigentliche Herzfrequenz. |

Genau genommen ist diese vom Puls zu unterscheiden (der Puls wird peripher gemessen, zum Beispiel: Radialispuls).

| | |
|---|---|
| BP | Blood Pressure |
| 122 lbs | 55.3 kg |
| HEENT | Head, Eyes, Ears, Nose, and Throat |
| NC/AT | normocephalic/atraumatic |
| EOMI | Extraocular Movements Intact |
| PERRLA | Pupils Equal, Round, Reactive to Light and Accommodation |
| JVD | Jugular Venous Distention |

Kernig's sign Kernig-Zeichen
Zeichen für Meningismus: es ist positiv, wenn bei 90° Hüftbeugung (z. B. Sitzen am Bettrand oder angewinkeltem Bein im Liegen) der Unterschenkel im Knie wegen beim Strecken auftretenden Schmerzen nicht vollständig gestreckt werden kann.

egophony = „e to a transition": der Pat. wird bei der Untersuchung der Lunge angewiesen "e" zu sagen, bei der Auskultation ist ein "a" zu hören., beipsielsweise bei einer Pneumonie (ähnlich der Bronchophonie). In Deutschland selten praktizierte Untersuchungstechnik.

SEM Systolic Ejection Murmur (systolisches Herzgeräusch)
ICS Inter-Costal Space
PMI Point of Maximum Impulse (Befund bei der Palpation, bezeichnet die Stelle, an der der Herzspitzenstoß am deutlichsten zu tasten ist. Normalerweise 5. ICR in den Medioclavicularlinie).
CVA Costovertebral Angle Tenderness (Nierenklopfschmerz)
DP Dorsalis Pedis
DTR Deep Tendon Reflexes

<u>Card 6</u>

Bitte beachten, dass hier mehrere Antwortmöglichkeiten als richtig markiert werden können.

<u>Card 8</u>

RR Respiratory Rate (Atemfrequenz)
WBC White Blood Count (Leukozytenzahl)
UO Urine Output

Creatinine 0.5mg/dL = 44.2 mmol/l

SD Standard Deviation (Standardabweichung)
MAP Mean Arterial Pressure (mittlerer arterieller Druck)

<u>Card 9</u>

CBC Complete Blood Count („Blutbild" incl. Erythrozyten- und Thrombozytenzahl)

*Chemistry Panel* umfasst folgende Laborparameter: Sodium (Natrium), Potassium (Kalium), Chloride, Carbon Dioxide (CO2), Albumin, Alkaline Phosphatase (AP), Bilirubin Direct, Bilirubin Total, Aspartate Transaminase (ASAT), Alanine Transaminase (ALAT), Blood Urea Nitrogen (BUN = Harnstoff), Total Protein, Calcium (Ca), Creatinine, Phosporus (Phosphat), Glucose.

UA — Urine Analysis (Urinstatus und Urinsediment, umfasst hier auch eine Gram-Färbung – letzteres in Deutschland nicht üblich).
CXR — Chest X-Ray (Röntgen Thorax)
FOBT — Faecal Occult Blood Test (“Hämoccult”)
PCP — Primary Care Physician (Hausarzt

<u>Card 10</u>

Plt — Platelets (Thrombozyten)
ECG — Electrocardiogram (EKG)
BUN — Blood Urea Nitrogen (Harnstoff)
Cre — Creatinine

<u>Card 12</u>

RBC — Red Blood Count (Erythrozytenzahl)
WBC — White Blood Count (Leukozytenzahl)

<u>Card 14</u>

cc — cubic centimetre ($cm^3$ = ml)
UTI — Urinary Tract Infection

<u>Card 15</u>

Deutsche Leitlinie Sepsis:
http://www.awmf.org/uploads/tx_szleitlinien/079-001l_S2k_Sepsis_Leitlinientext_01.pdf

<u>Card 16</u>

Ticarcillin — Breitspektrum-Penicillin (in Deutschland üblicherweise nicht eingesetzt, in *Roter Liste* nicht geführt)

Foley catheter — Harnblasenkatheter (benannt nach Frederic Foley)

AA&Ox4 — Awake, Alert, and Oriented to Person, Place, Time, and Events

<u>Card 17</u>

In Deutschland entspricht der link zu *advance care planning* im Text etwa diesem link zum Bundesministerium für Justiz:
http://www.bmj.de/DE/Buerger/gesellschaft/Patientenverfuegung/patientenverfuegung_node.html

Bei uns wäre es in dieser Situation sicherlich nicht die Aufgabe eines PJ-Studenten, ein Gespräch über eine Patientenverfügung mit Angehörigen oder dem Patienten zu besprechen. Dies sollte ein erfahrenerer Arzt übernehmen, nach Möglichkeit freilich unter Begleitung des PJ-Studenten.

Card 18

Bei Hämatochezie ist, wenn eine Perforation ausgeschlossen werden konnte (Röntgen Abdomen), eine Koloskopie gerechtfertigt, bzw. - wenn eine Reinigung des ganzen Kolon durch orthograde Darmlavage nicht rasch und sicher genug möglich ist - eine Sigmoido-/Rektoskopie nach vorbereitendem Klysma/Einlauf. Sicherlich auch aus Kostengründen (jede endoskopische Diagnostik ist in den USA wesentlich teurer als in Deutschland!) wird die Endoskopie in den USA wesentlich seltener eingesetzt und eine radiologische Diagnostik favorisiert (Problem: Strahlenbelastung!).

Card 19

Leider ist nicht ersichtlich, in welcher Technik die Abdomen-Übersicht angefertigt wurde. Die Aufnahme sieht aus wie eine Liegendaufnahme (a.p.). Eine Aufnahme im Stehen wird bei dem Patienten nicht möglich gewesen sein. Zum Ausschluss einer Perforation wäre aber eine Aufnahme in Linksseitenlage erforderlich (Luftsichel zwischen Leber und Bauchwand), ebenso zur Darstellung von Spiegeln bei einem Ileus.

Hämoglobin 10 g/dL = 6.21 mmol/l
8 g/dL = 4.96 mmol/l

Zur Indikationsstellung zur Gabe von Erythrozytenkonzentraten vgl. Leitlinie der Bundesärztekammer:

<table>
<tr><td colspan="4">Zur Indikationsstellung einer Erythrozytentransfusion wird die individuelle Berücksichtigung der Kriterien Hb-Konzentration, Kompensationsfähigkeit und Risikofaktoren des Patienten empfohlen:</td></tr>
<tr><th>Hb-Bereich</th><th>Kompensationsfähigkeit/Risikofaktoren</th><th>Transfusion</th><th>Bewertung</th></tr>
<tr><td>≤ 6 g/dl (≤ 3,7 mmol/l)</td><td>-</td><td>ja*</td><td>1 C+</td></tr>
<tr><td rowspan="3">> 6–8 g/dl (3,7–5,0 mmol/l)</td><td>Kompensation adäquat, keine Risikofaktoren</td><td>nein</td><td>1 C+</td></tr>
<tr><td>Kompensation eingeschränkt, Risikofaktoren vorhanden (z.B. KHK, Herzinsuffizienz, cerebrovaskuläre Insuffizienz)</td><td>JA</td><td>1 C+</td></tr>
<tr><td>Hinweise auf anämische Hypoxie (Physiologische Transfusionstrigger: z.B. Tachykardie, Hypotension, EKG-Ischämie, Laktazidose)</td><td>ja</td><td>1 C+</td></tr>
<tr><td>8–10 g/dl (5,0–6,2 mmol/l)</td><td>Hinweise auf anämische Hypoxie (Physiologische Transfusionstrigger: z.B. Tachykardie, Hypotension, EKG-Ischämie, Laktazidose )</td><td>ja</td><td>2 C</td></tr>
<tr><td>> 10 g/dl (≥ 6,2 mmol/l)</td><td>-</td><td>nein**</td><td>1 A</td></tr>
<tr><td colspan="4">Merke!<br>Die Hämoglobinkonzentration allein ist kein adäquates Maß des $O_2$-Angebots.<br>Bei Hypovolämie gibt der Hämatokrit den Erythrozytenmangel nicht korrekt wieder.<br>Individuelle Faktoren können eine von den Empfehlungen abweichende Indikationsstellung erforderlich machen.</td></tr>
</table>

aus **http://www.bundesaerztekammer.de/downloads/Querschnittsleitlinie_Gesamtdokument-deutsch_07032011.pdf**

## Card 20

| | |
|---|---|
| NPO | nil per os (nüchtern lassen) |
| Hb | 8.1 g/dL = 5.0 mmol/l |

Court appointed guardian — vom Gericht bestellter Betreuer
Durable Power Of Attorney — "dauerhafte Bevollmächtigung" (Vorsorgevollmacht)

Nach deutschem Recht hat die Familie kein Mitbestimmungsrecht auf die durchzuführenden medizinischen Maßnahmen, es sei denn, ein Angehöriger ist in einer *Vorsorgevollmacht* vom Patienten im Voraus hierzu berufen worden. Bei der Einbeziehung der Angehörigen in die Entscheidungsfindung geht es um die Ermittlung des mutmaßlichen Willens des nicht entscheidungsfähigen Patienten.
Im Gegensatz dazu wird in einer *Patientenverfügung* im Voraus festgelegt, wie der Verfügende nach seinem Willen als Patient ärztlich behandelt werden möchte, wenn er nicht mehr in der Lage ist, selber darüber zu entscheiden.

## Card 21

| | |
|---|---|
| STAT | statim (sofort) |
